# Heparin-Functionalized Adsorbents Eliminate Central Effectors of Immunothrombosis, including Platelet Factor 4, High-Mobility Group Box 1 Protein and Histones

**DOI:** 10.3390/ijms23031823

**Published:** 2022-02-05

**Authors:** Marie Ebeyer-Masotta, Tanja Eichhorn, René Weiss, Vladislav Semak, Lucia Lauková, Michael B. Fischer, Viktoria Weber

**Affiliations:** 1 Center for Biomedical Technology, Department for Biomedical Research, Danube University Krems, 3500 Krems, Austria; marie.ebeyer-masotta@donau-uni.ac.at (M.E.-M.); tanja.eichhorn@donau-uni.ac.at (T.E.); rene.weiss@donau-uni.ac.at (R.W.); vladislav.semak@donau-uni.ac.at (V.S.); lucia.krajcik-laukova@donau-uni.ac.at (L.L.); michael.fischer@donau-uni.ac.at (M.B.F.); 2 Clinic for Blood Group Serology and Transfusion Medicine, Medical University of Vienna, 1090 Vienna, Austria

**Keywords:** adsorption, COVID-19, extracellular vesicles, heparin, immunothrombosis, neutrophil extracellular traps, platelet factor 4, platelets, sepsis

## Abstract

Inflammation and thrombosis are closely intertwined in numerous disorders, including ischemic events and sepsis, as well as coronavirus disease 2019 (COVID-19). Thrombotic complications are markers of disease severity in both sepsis and COVID-19 and are associated with multiorgan failure and increased mortality. Immunothrombosis is driven by the complement/tissue factor/neutrophil axis, as well as by activated platelets, which can trigger the release of neutrophil extracellular traps (NETs) and release further effectors of immunothrombosis, including platelet factor 4 (PF4/CXCL4) and high-mobility box 1 protein (HMGB1). Many of the central effectors of deregulated immunothrombosis, including activated platelets and platelet-derived extracellular vesicles (pEVs) expressing PF4, soluble PF4, HMGB1, histones, as well as histone-decorated NETs, are positively charged and thus bind to heparin. Here, we provide evidence that adsorbents functionalized with endpoint-attached heparin efficiently deplete activated platelets, pEVs, PF4, HMGB1 and histones/nucleosomes. We propose that this elimination of central effectors of immunothrombosis, rather than direct binding of pathogens, could be of clinical relevance for mitigating thrombotic complications in sepsis or COVID-19 using heparin-functionalized adsorbents.

## 1. Introduction

Immunothrombosis denotes the concurrent activation of the innate immune and coagulation systems to entrap and eliminate pathogens in the circulation [1]. Deregulated immunothrombosis, leading to microthrombus formation in the capillaries and to loss of endothelial barrier function, is a hallmark of sepsis and a major pathologic event in COVID-19 [2,3], where arterial, microvascular and venous thrombosis exacerbate organ injury and are related to poor prognosis [4,5].

Platelets are effectors in mediating hemostasis and thrombosis. As the second most abundant cells in the circulation, they are important sentinels of bacterial or viral infection and mediate the innate immune response by modulating leukocyte migration [6,7,8], leukocyte recruitment to thrombi [9], NET formation [10], monocyte expression of tissue factor (TF) [11,12], as well as secretion of chemokines, including PF4 [13]. Upon activation, platelets readily release extracellular vesicles, which support coagulation via their exposure of phosphatidylserine [14,15] and exert roles in immunomodulation [16,17] by carrying a molecular cargo, including cytokines [18] and lipid mediators [19], as well as damage-associated molecular pattern molecules (DAMPs), such as high-mobility group box 1 protein [20].

PF4 is an abundant platelet α-granule chemokine released during platelet activation. In addition to this soluble form, PF4 is displayed on the surface of activated platelets and of pEVs. PF4 levels are strongly elevated in sepsis [21,22] and COVID-19 [23]. The binding of PF4 to heparin and to polyanionic cell surface glycosaminoglycans, including heparan sulfate, is well-established [24,25]. Clinically, this interaction can result in heparin-induced thrombocytopenia (HIT), where complexes of PF4 and heparin induce antibody formation with subsequent platelet activation and induction of a pro-thrombotic state. A similar condition, vaccine-induced immune thrombotic thrombocytopenia (VITT), with high levels of anti-PF4 antibodies, has recently been described following administration of the ChAdOx1 nCoV-19 adenoviral vector vaccine [26,27].

HMGB1, a highly conserved, bi-polar, non-histone nuclear DNA-binding protein, acts as DAMP and induces inflammation upon its release by necrotic cells or its active secretion by stressed cells. Platelets express HMGB1, which is displayed on their surface as well as released into the extracellular space upon platelet activation [28,29]. Plasma levels of HMGB1 are upregulated in conditions associated with abnormal coagulation, including sepsis [30] and COVID-19 [31]. The diverse biological functions of HMGB1 resemble those of activated platelets, such as the induction of NET formation [32] and microvascular thrombosis [33], identifying platelet-derived HMGB1 as a link between inflammation and thrombosis. Recent studies have indicated that activated platelets are a major source of HMGB1 within thrombi, suggesting that it may constitute a target for antithrombotic therapy [34]. Interaction of HMGB1 with heparin induces a conformational change and decreases its affinity for its main receptor for advanced glycation end products (RAGE) [35].

Histones are highly basic DNA-binding proteins. By forming a complex with DNA, the nucleosome, histones H2A, H2B, H3 and H4 facilitate higher-order chromatin compaction. Similarly to HMGB1, histones act as DAMPs when released from necrotic cells or from activated neutrophils during NET formation. Histone levels in septic patients are significantly increased and appear to cause cellular injury in a TLR4-dependent manner [35,36]. Likewise, pronounced NET formation [37,38] and elevated levels of citrullinated histone H3 have recently been found in patients suffering from COVID-19 and were correlated with disease severity [39,40].

Next to drug therapies for the treatment of COVID-19, which are still awaiting confirmatory evidence, extracorporeal blood purification has been suggested as a supportive measure [41,42,43], and emergency use authorization for extracorporeal blood purification devices in COVID-19 was granted by the US Food and Drug Administration (FDA) in April 2020. One of these extracorporeal devices, the Seraph-100 Microbind Affinity Blood Filter, consists of ultra-high molecular weight polyethylene beads with endpoint-attached heparin [44]. While this extracorporeal approach is currently mainly explored with a view of depleting pathogens from the circulation, which are claimed to bind to immobilized heparin in a similar manner to their interaction with heparan sulfate on the cell surface, we propose and provide evidence that heparin-immobilized adsorbents may exert beneficial effects by binding and depleting activated PF4^+^ platelets and PF4^+^ pEVs, as well as HMGB1 and histones/nucleosomes, thereby contributing to the alleviation of immunothrombosis at multiple levels.

## 2. Results

### 2.1. Heparinized Adsorbents Efficiently Deplete PF4, Histones/Nucleosomes and HMGB1

**Figure 1:**
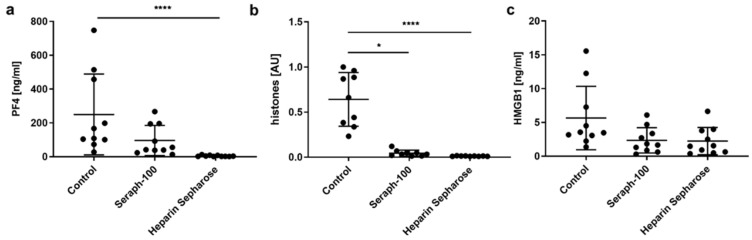
Depletion of PF4, histones/nucleosomes and HMGB1 by Seraph-100 and Heparin Sepharose. Plasma samples from sepsis patients were incubated with 10 vol% of adsorbent for 60 min at 37 °C and the remaining concentrations for (**a**) PF4, (**b**) histones/nucleosomes and (**c**) HMGB1 were determined. Plasma samples incubated without adsorbent served as controls. Data are presented as mean ± standard deviation (Kruskal–Wallis test followed by Dunn’s multiple comparisons; *n* = 9 for nucleosomes, *n* = 10 for HMGB1 and PF4; * *p* < 0.05, **** *p* < 0.0001). Results for histones/nucleosomes are given as arbitrary units (AU), which were calculated as a ratio of the highest optical density (OD) value and the OD value of individual samples.

Incubation of septic plasma samples with Seraph-100 and Heparin Sepharose, resulted in efficient depletion of PF4 and histones/nucleosomes. Using 10 vol% of adsorbent, both the PF4 and histones/nucleosomes were reduced to levels below the limit of detection with Heparin Sepharose and lowered to 50% and 11% of their initial levels for Seraph-100 (Figure 1), respectively, while their levels remained unaffected by treatment with non-functionalized Sepharose, as compared to the untreated control. HMGB1 levels were lowered by both heparin-functionalized adsorbents, resulting in a reduction of 58.9 ± 14.7% for Seraph-100 and 63.1 ± 12.2% for Heparin Sepharose, as compared to the untreated control (*n* = 10; samples from 10 different patients).

### 2.2. Platelet Activation and Release of pEVs in Response to TRAP-6 Are Dose-Dependent

**Figure 2:**
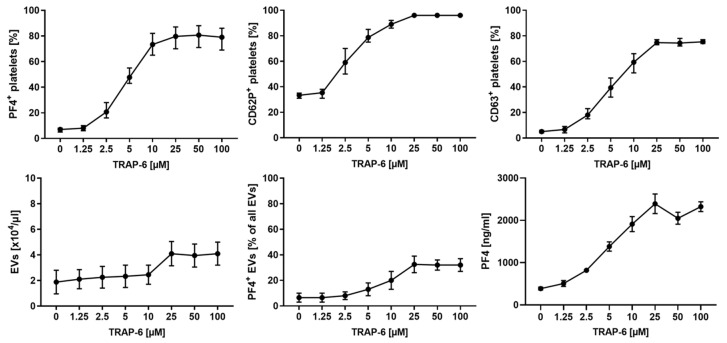
Platelet activation with TRAP-6. Platelets suspended in SSP^+^ anticoagulated with ACD-A 1:12 (233 ± 41 × 10^3^ platelets/µL) were activated for 10 min at room temperature with 1.25, 2.5, 5, 10, 25, 50 or 100 µM TRAP-6, or were left untreated. Platelets and pEVs were analyzed by flow cytometry, using CD41 as platelet marker and CD62P (P-selectin), PF4 (platelet factor 4) and CD63 (lysosomal membrane glycoprotein) as platelet activation markers. Annexin5 (Anx5) was used as a marker for phosphatidylserine exposed on EVs. Platelets were identified as CD41^+^ cells, and EVs were defined as CD41^+^ Anx5^+^ events in the EV gate, as described in the methods section. Soluble PF4 was quantified by ELISA. Data are given as mean ± range (*n* = 3 for platelet analysis; *n* = 2 for EVs and PF4 analysis). For comparison, platelets were activated using thrombin (Supplementary Figure S1).

To find an optimal concentration of thrombin receptor activator peptide-6 (TRAP-6) for platelet activation, we incubated platelets with increasing concentrations of TRAP-6, as described in the methods section. Expression of CD62P (P-selectin), PF4 (released from α-granules) and CD63 (released from dense granules) increased in a dose-dependent manner, and reached a plateau at 25 µM of TRAP-6 for all activation markers. Platelet activation was accompanied by an increasing release of pEVs, which also reached a plateau at 25 µM TRAP-6 (Figure 2). A concentration of 25 µM TRAP-6 was thus used to activate platelets for the re-circulation experiments described below. 

### 2.3. TRAP-6 Activated Platelets and pEVs Are Bound by Heparin-Functionalized Adsorbents

**Figure 3:**
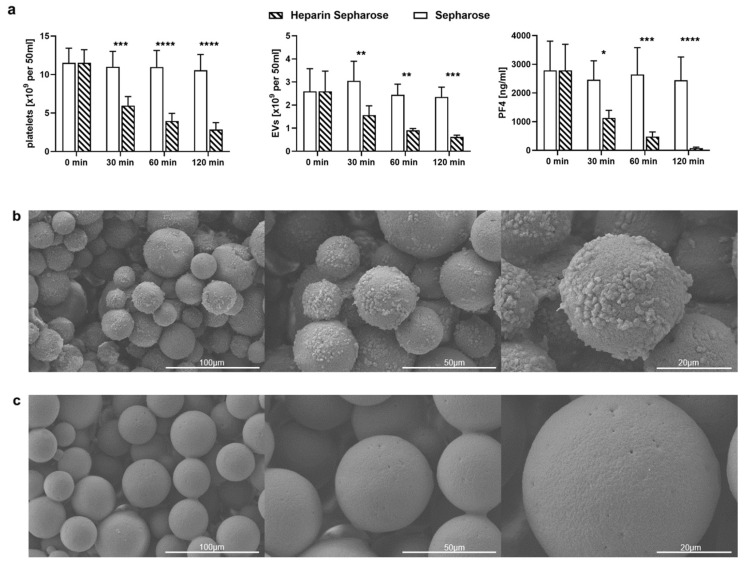

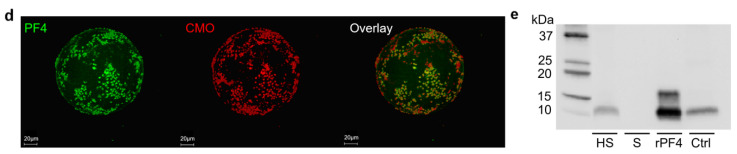
Binding of activated platelets and pEVs by Heparin Sepharose. Platelets (50 mL suspension containing 244 ± 42 × 10^3^ platelets/µL) were activated with 25 µM TRAP-6 and re-circulated over columns containing 5 mL Heparin Sepharose or non-functionalized Sepharose. Samples were drawn immediately after platelet stimulation with TRAP-6 (25 µM, 10 min at room temperature), and after 30, 60 and 120 min of re-circulation. Platelets and EVs were characterized by flow cytometry, and PF4 was quantified by ELISA. (**a**) Depletion of platelets, pEVs and soluble PF4 upon re-circulation over Heparin Sepharose and non-functionalized Sepharose. Data are given as mean ± standard deviation (*n* = 6 for Heparin Sepharose and *n* = 3 for non-functionalized Sepharose). Repeated-measure two-way ANOVA, followed by Sidak’s multiple comparisons test, was performed to assess differences between Heparin Sepharose and non-functionalized Sepharose at individual time points (* *p* < 0.05, ** *p* < 0.01, *** *p* < 0.001, **** *p* < 0.0001). After 120 min of re-circulation, columns were disassembled, and (**b**) Heparin Sepharose beads as well as (**c**) Sepharose beads were analyzed by scanning electron microscopy. (**d**) Confocal laser scanning microscopy showing PF4^+^ platelets adsorbed to Heparin Sepharose beads. Platelets were stained using mouse anti-PF4 as a primary antibody and AF488-conjugated goat anti-mouse IgG as a secondary antibody (green), as well as CellMask Orange (CMO, red). (**e**) Western blotting was performed on protein fractions eluted from Heparin Sepharose beads after 120 min of re-circulation. HS, Heparin Sepharose; S, non-functionalized Sepharose; rPF4, recombinant PF4; Ctrl, activated platelet suspension. 20 µg of protein were loaded per lane.

To assess the binding of activated platelets under dynamic conditions, they were re-circulated over adsorbent columns packed with Heparin Sepharose or with non-functionalized Sepharose, as described in the methods section, revealing that activated platelets adhered to Heparin Sepharose to a significantly higher extent than to non-functionalized Sepharose. Platelet counts were reduced by 75% after 120 min by Heparin Sepharose, whereas they remained stable in the circuit containing non-functionalized Sepharose (Figure 3a). This was further supported by scanning electron microscopy of the adsorbent beads after re-circulation, which showed that Heparin Sepharose beads were almost entirely covered by platelets, while Sepharose beads remained uncovered (Figure 3b,c). According to confocal microscopy, platelets bound to Heparin Sepharose beads stained positive for PF4 (Figure 3d), and the presence of PF4 on Heparin Sepharose was further confirmed by Western blotting of protein fractions eluted from the adsorbent beads (Figure 3e).

### 2.4. Heparinized Adsorbents Preferentially Bind PF4^+^ Platelets and pEVs

**Figure 4:**
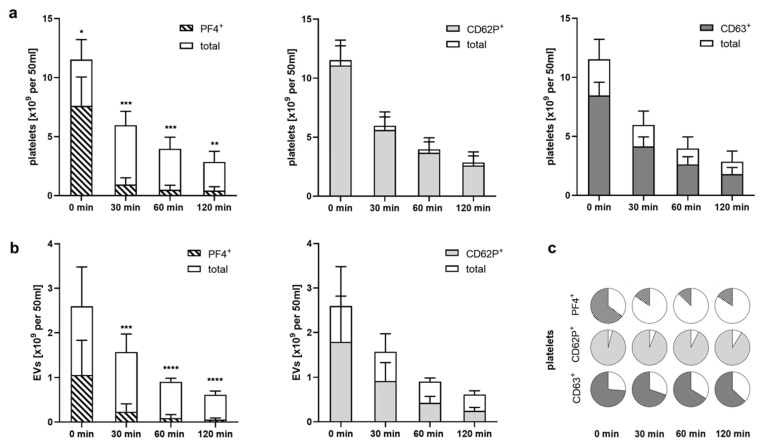
Preferential binding of PF4^+^ platelets to Heparin Sepharose. Data shown in this figure were obtained by re-circulation of activated platelets over Heparin Sepharose, as shown in Figure 3. (**a**) Decrease in platelets exposing PF4^+^, CD62P^+^ and CD63^+^ in relation to the decrease in total platelet counts upon re-circulation of activated platelets over Heparin Sepharose; (**b**) preferential depletion of PF4^+^ pEVs over CD62P^+^ pEVs. Data are given as mean ± standard deviation (*n* = 6); (**c**) percentages of PF4^+^, CD62P^+^ and CD63^+^ platelets over the course of the experiment, illustrating the preferential binding of PF4^+^ platelets. * *p* < 0.05, ** *p* < 0.01, *** *p* < 0.001, **** *p* < 0.0001.

Comparing the rate of platelet depletion during re-circulation of activated platelets over Heparin Sepharose, we found that PF4^+^ platelets were preferentially bound, whereas platelets that were positive for CD62P or CD63, but did not carry PF4, were less prone to adsorption. At the onset of re-circulation, 66%, 95% and 73% of all platelets stained positive for PF4, CD62P and CD63, respectively. After 120 min of re-circulation, only 15% of the platelets remaining in the circulation were PF4^+^, whereas 91% and 64% were CD62P^+^ or CD63^+^ (Figure 4). The same was found for PF4^+^ pEVs, which were preferentially depleted, as well.

## 3. Discussion

Deregulated immunothrombosis is caused by a maladapted response of the innate immune system to infection. The contribution of the complement/neutrophil/tissue factor axis to immunothrombosis is well recognized [45,46,47]. Deregulated complement activation drives inflammation by enhancing neutrophil activation and recruitment to the infected lungs, and by promoting TF expression, leading to microvascular thrombosis and endothelial dysfunction. Excessive release of NETs decorated with TF and histones further promotes thrombosis and tissue damage [8].

Next to this complement/neutrophil/tissue factor axis, platelets are the main effectors of immunothrombosis. Beyond their hemostatic activity, activated platelets trigger NET formation and modulate cellular functions of adjacent immune cells [48]. This study, among others, has provided evidence that activated platelets, as well as pEVs, induce a shift towards inflammatory CD16^+^ monocyte subsets [49,50,51], which has been implicated in disease severity in sepsis as well as in COVID-19 [52]. Furthermore, numerous studies have consistently demonstrated platelet hyperactivation in COVID-19 patients, especially in those with severe disease [2].

Many of the central effectors of deregulated immunothrombosis, including PF4^+^ platelets and pEVs, soluble PF4, HMGB1, histones, as well as histone-decorated NETs, share an affinity for heparin. Their binding to heparin is mainly mediated via nonspecific electrostatic interactions, e.g., in the case of PF4 and histones, which are basic proteins and interact with the negatively charged sulfate groups and uronic acid residues in the heparin chain. Likewise, the binding site of HMGB1 to heparin has been mapped to a basic loop region connecting the A-box and B-box domains of the molecule [53].

This well-established affinity between effectors of immunothrombosis and heparin led us to assess the ability of adsorbents functionalized with endpoint-attached heparin to deplete these factors from the circulation. As a proof of principle, we investigated the ability of commercial Heparin Sepharose to deplete soluble PF4, as well as PF4^+^ platelets and pEVs from activated platelet concentrates in re-circulation experiments in vitro. In line with our hypothesis, the re-circulation of activated platelets over Heparin Sepharose resulted in a significant depletion of platelets and pEVs, as well as soluble PF4, over time. Flow cytometry revealed a disproportionately strong decrease in PF4^+^ platelets and PF4^+^ pEVs, and, consistently, the entirety of the platelets adhering to the adsorbent beads stained positive for PF4, suggesting there was PF4-mediated binding of activated platelets to heparin-functionalized adsorbents. To extend our hypothesis, we performed ex vivo batch experiments with plasma from sepsis patients. Using the clinically approved adsorbent Seraph-100 in addition to Heparin Sepharose, we observed efficient depletion of soluble PF4 and histones/nucleosomes, as well as HMGB1.

Seraph-100 has been developed following a biomimetic approach with the underlying assumption that bacterial, as well as viral pathogens, can bind to immobilized heparin in a similar way to the way they interact with heparan sulfate on cellular surfaces. So far, Seraph-100 has been exploited for its ability to deplete carbapenem-resistant Enterobacteriaceae in vitro, where it yielded promising results [54]. The first clinical case reports on the capacity of the Seraph-100 Microbind Affinity Blood Filter to eliminate *Staphylococcus aureus* from the blood stream have been published recently [55].

There is evidence that the viral load is associated with increased disease severity and mortality in COVID-19 [56], and that the spike protein of SARS-CoV-2 binds to heparan sulfate and heparin through its receptor-binding domain [57,58]. It was therefore obvious to consider the application of Seraph-100 as a supportive therapy in COVID-19. The Seraph-100 Microbind Affinity Blood Filter obtained emergency use authorization for COVID-19 by the FDA in 2020, followed by a case series assessing its use in SARS-CoV-2 infected patients early in critical illness [59], however, this study did not collect data on virus elimination from the circulation. A follow-up study provided evidence that treatment with Seraph-100 decreased the SARS-CoV-2 nucleocapsid protein in critically ill patients [60]; however, the effect on clinically relevant outcome parameters remains open.

As evident as it may seem, the concept of focusing on the elimination of pathogens from the circulation by using heparin-immobilized adsorbents appears challenging, based on the following considerations: first, a multitude of pathogens can cause blood stream infection, and their affinity for heparin varies over a wide range; second, pathogen loads of typically 1–10 colony-forming units per mL have been reported in sepsis [61], and it is difficult to conceive that a specific elimination of pathogens from whole blood can occur at such low abundance; third, intracellular pathogens would not be amenable for adsorption, while in fact both bacterial and viral pathogens can persist in immune cells [62], and macrophages have been reported to act as vectors of infection that contribute to the dissemination of SARS-CoV-2 [63,64].

Still, the concept of using extracorporeal therapies with immobilized heparin could be beneficial beyond the elimination of pathogens from the circulation, namely in terms of targeting effectors of immunothrombosis. Many of these mediators, including PF4, bind to heparin, and it is likely that their excessive release is a major cause of heparin resistance observed patients suffering from severe COVID-19. The data presented here provide evidence for the efficient depletion of these factors, since PF4 and HMGB1, as well as histones/nucleosomes, were efficiently eliminated by heparinized adsorbents in our study. This is particularly relevant since both PF4 and HMGB1 are strong inducers of NET formation [65,66], and the role of NETs in SARS-CoV-2 pulmonary pathophysiology has been impressively shown [8]. Furthermore, elevated levels of PF4 and platelet-neutrophil aggregates have been observed in COVID-19 patients. PF4 released from activated platelets binds to NETs and renders them resistant to DNase [67], and further PF4 is released from platelets trapped inside NETs, so that the PF4/NET positive feedback loop initiates and sustains a coagulative cascade [8]. While we did not assess the depletion of NETs by heparinized matrices, the binding of PF4, PF4^+^ platelets and HMGB1, as well as histones, all of which are components of NETs, strongly suggests that heparin-functionalized adsorbents are capable of eliminating NETs, and that the use of these adsorbents in extracorporeal therapies can be a beneficial clinical intervention to reduce excessive NET formation.

## 4. Materials and Methods

### 4.1. Chemicals and Reagents

Phosphate-buffered saline (PBS) was purchased from Life Technologies (Paisely, UK). Physiological sodium chloride solution was obtained from Fresenius Kabi (Graz, Austria). Anticoagulant citrate dextrose solution A (ACD-A; 22.0 g/L trisodium citrate, 24.5 g/L glucose monohydrate, 7.3 g/L citric acid) was obtained from Terumo BCT (Zouventem, Belgium). Platelet storage medium SSP^+^ (3.18 g/L trisodium citrate dihydrate, 4.42 g/L sodium acetate trihydrate, 1.05 g/L sodium dihydrogen phosphate dihydrate, 3.05 g/L disodium hydrogen phosphate, 0.37 g/L potassium chloride, 0.30 g/L magnesium chloride hexahydrate, 4.05 g/L sodium chloride; pH 7.2) was purchased from Macopharma (Tourcoing, France). TRAP-6 was obtained from Bachem (Bubendorf, Switzerland). Glutaraldehyde was purchased from Carl Roth (Karlsruhe, Germany). Heparin was obtained from Gilvasan Pharma (Vienna, Austria). Bovine serum albumin was obtained from Sigma-Aldrich (Saint Louis, MO, USA).

### 4.2. Plasma and Platelet Concentrates

Plasma samples from sepsis patients were obtained from the University Clinic St. Poelten, Austria, as approved by the local ethics committee on 12 March 2013 (GS4-EK-3/082-2012) and in accordance with the Declaration of Helsinki. Written informed consent was obtained from all donors. All samples contained sodium citrate as an anti-coagulant and were stored at −80 °C until further use.

Platelet concentrates from healthy volunteer donors were generated by single donor platelet apheresis at the Clinic for Blood Group Serology and Transfusion Medicine, Medical University of Vienna, Vienna, Austria, using a Trima Accel R automated blood collection system (Version 5.0, Terumo BCT). The study was approved by the local ethics committee on 16 February 2016 (ECS2177/2015), and written informed consent was obtained from all donors. Platelets were collected into polyolefin bags containing 80% SSP^+^ medium and 20% plasma.

### 4.3. Adsorbents

Two heparin-functionalized adsorbents were used in this study. Heparin Sepharose CL-6B (Cytiva, Uppsala, Sweden) is composed of 6% cross-linked agarose beads, functionalized with porcine heparin. The Seraph-100 Microbind Affinity Blood Filter (ExThera Medical, Martinez, CA, USA), which is clinically approved for therapeutic apheresis, contains ultrahigh-molecular weight polyethylene beads with endpoint-attached heparin [68]. Non-functionalized Sepharose CL-6B (Cytiva, Uppsala, Sweden) was used as a control. Chemical composition of the carrier beads and average particle size, as well as ligand density for all adsorbents used in this study, are given in Table 1. All adsorbents were extensively washed with physiological saline solution prior to use.

### 4.4. Adsorption of PF4, Histones/Nucleosomes and HMGB1

**Figure 5:**
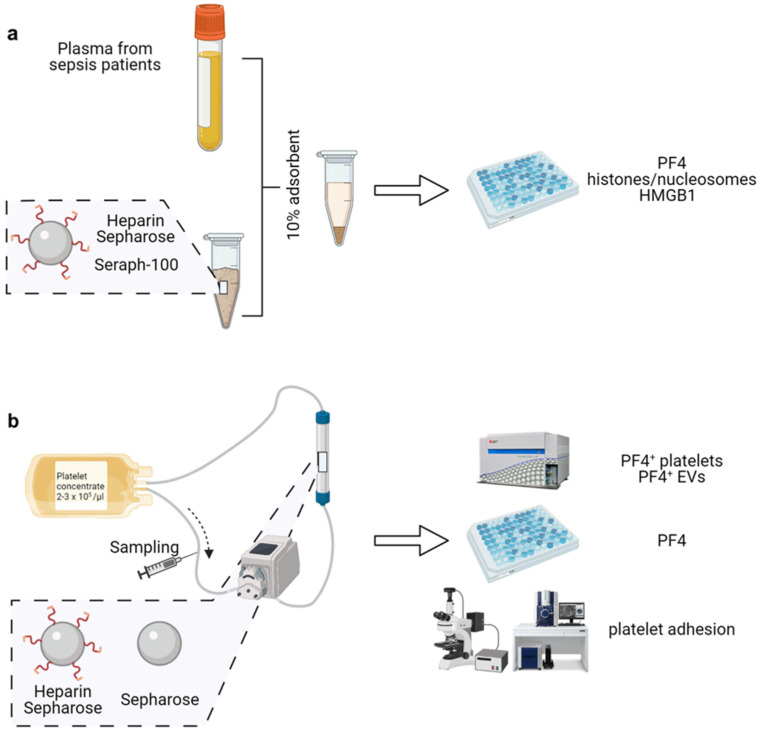
Experimental set-up. (**a**) Batch experiments. Plasma from sepsis patients (*n* = 10) was incubated with 10 vol% of Seraph-100 or Heparin Sepharose, or with non-functionalized Sepharose as a control, as described in the methods section. Treated plasma was collected, and soluble PF4, histones/nucleosomes and HMGB1 were quantified by ELISA; (**b**) re-circulation experiments. The depletion of activated platelets and pEVs by the adsorbents was assessed by circulating activated platelets (2–3 × 10^5^/µL in SSP^+^ medium anticoagulated with ACD-A 1:12; total volume 50 mL) over adsorbent columns packed with 5 mL Heparin Sepharose or with non-functionalized Sepharose as a control, at a flow rate of 1 mL/min. Samples were drawn immediately after platelet stimulation (baseline value) and after 30, 60 and 120 min of re-circulation. Figure created with BioRender.com (accessed on 20 December 2021).

To assess the ability of heparin-functionalized adsorbents to deplete PF4, histones/nucleosomes and HMGB1, plasma samples from 10 sepsis patients were incubated with 10 vol% of Heparin Sepharose or Seraph-100 for 60 min at 37 °C with gentle rotation, or with non-functionalized Sepharose as a control (Figure 5a). Following incubation, the adsorbent beads were pelleted by centrifugation (400 g, 5 min), plasma was collected, aliquoted and stored at −20 °C until further analysis.

### 4.5. Quantification of PF4, Histones/Nucleosomes and HMGB1

Soluble PF4 was quantified by ELISA (R&D Systems, Minneapolis, MN, USA). Histones/nucleosomes were quantified using the Cell Death ELISA (Roche, Mannheim, Germany). Since this assay does not discriminate between nucleosomes and core histones, both terms are used jointly throughout the manuscript. HMGB1 levels were analyzed by ELISA (IBL International, Hamburg, Germany).

### 4.6. Platelet Activation by TRAP-6

Platelets (2–3 × 10^5^/µL in SSP^+^ medium anticoagulated with ACD-A 1:12; final citrate concentration 10.9 mM) were activated with increasing concentrations of TRAP-6 (1.25, 2.5, 5, 10, 25, 50, 100 µM) for 10 min at room temperature in the dark. Platelet activation was assessed by flow cytometry as described below.

### 4.7. Flow Cytometric Characterization of Platelets and Platelet-Derived Extracellular Vesicles

Platelets and pEVs were characterized by flow cytometry using a CytoFLEX LX device (Beckman Coulter, Brea, CA, USA) equipped with 405 nm, 488 nm, 561 nm and 631 nm lasers. For staining, samples were diluted 1:100 in Annexin5 (Anx5) binding buffer (BD Biosciences San Jose, CA, USA). Aliquots (100 μL each) of the diluted samples were incubated for 15 min at room temperature in the dark with a combination of PC7-conjugated anti-CD41 (Beckman Coulter) as a platelet marker, and FITC-conjugated anti-CD62P (Beckman Coulter) and PE-conjugated anti-PF4 (R&D Systems), as well as AF647-conjugated anti-CD63 (BioLegend, San Diego, CA, USA) as platelet activation markers. APC-conjugated Anx5 (BD Biosciences) was used as a marker for EVs exposing phosphatidylserine. All fluorochrome conjugates, and the respective antibody clones, are listed in Table 2. To remove any precipitates, fluorochrome conjugates were centrifuged at 18,000 *g* for 10 min at 4 °C prior to use.

Calibration of the flow cytometer was performed with fluorescent silica beads (1 μm, 0.5 μm, 0.3 μm, 0.1 μm; excitation/emission 485/510 nm; Kisker Biotech, Steinfurt, Germany). The triggering signal for EVs was set to the violet side scatter (405 nm), and the EV gate was set below the 1 µm bead cloud as previously described [14,69,70]. For platelet characterization, the triggering signal was set to the 488 nm side scatter (Supplementary Figure S2).

Prior to analysis, stained samples were diluted 1:5 in sterile-filtered Anx5 binding buffer (BD Biosciences). Platelets were identified as CD41^+^ cells, and pEVs were identified as CD41^+^ Anx5^+^ events in the EV gate. Acquisition was performed for 2 min at a flow rate of 10 µL/min. Data were analyzed using the Kaluza Software (Beckman Coulter).

### 4.8. Re-Circulation of Activated Platelets over Adsorbent Columns

To assess the depletion of activated platelets and pEVs by the adsorbent polymers, platelets (2–3 × 10^5^/µL in SSP^+^ medium anticoagulated with ACD-A 1:12; total volume 50 mL) activated with 25 µM TRAP-6 were circulated over columns (1.3 cm × 3.8 cm) packed with 5 mL Heparin Sepharose or with non-functionalized Sepharose, at a flow rate of 1 mL/min using medical grade tubing sets and a hemodialysis roller pump (Fresenius Medical Care, Bad Homburg, Germany; Figure 5b). Samples were drawn immediately after platelet stimulation with TRAP-6 (baseline value), and after 30, 60 and 120 min of re-circulation. Platelets were quantified using a blood cell counter (Sysmex KX-21 N, Neumuenster, Germany), and PF4 was quantified by ELISA as described above. Flow cytometric characterization of platelets and pEVs was performed as described above.

### 4.9. Scanning Electron Microscopy

Platelet adhesion to the adsorbent beads was assessed by scanning electron microscopy. After 120 min of re-circulation (see above), adsorbent columns were washed with 50 mL of saline solution at a flow rate of 1 mL/min, and adsorbent beads from different areas of the column were collected. The beads were treated with 2.5% glutaraldehyde with gentle rotation (60 min, room temperature), dehydrated using an ethanol gradient (0% to 99.8%), dried, sputter-coated with gold (30 mA for 10 min; Q150R ES, Quorum Technologies, Laughton, UK) and analyzed with a FlexSEM-1000 scanning electron microscope (Hitachi, Tokyo, Japan).

### 4.10. Immunofluorescence Staining and Confocal Microscopy

Adhesion of PF4^+^ platelets to the adsorbent beads was visualized by confocal microscopy. Aliquots of 50 µL of fixed adsorbent beads were incubated under gentle rotation overnight at 4 °C with 500 µL of anti-PF4 (Santa Cruz Biotechnology, Dallas, TX, USA) in PBS containing 1% bovine serum albumin at a final antibody concentration of 2 µg/mL. After extensive washing with PBS containing 0.1% Tween 100 (Sigma-Aldrich), the beads were incubated for 60 min with a secondary anti-mouse antibody conjugated with AlexaFluor 488 (1:200; Jackson Immunoresearch, Ely, UK) and for 30 min with CellMask Orange (CMO; 1:1000; ThermoFisher Scientific, Waltham, MA, USA). Beads were washed twice with PBS containing 0.1% Tween 100, followed by three washing steps with saline solution (Fresenius Kabi), and one step with distilled H_2_O to avoid NaCl crystal formation during drying. Samples were mounted with Fluoromount aqueous mounting medium (Sigma-Aldrich) on high-precision microscope cover glasses (1.5H, Marienfeld, Lauda-Königshofen, Germany). Fluorescent images of the stained beads were acquired with a confocal laser scanning microscope (TCS SP8, Leica, Mannheim, Germany) using a 63× glycerol objective (numerical aperture 1.3). The 3D images of the beads were obtained by Z-stack imaging (acquisition of 718 Z-stack steps; step size: 0.34 µm; resolution: 512 × 512). Image analysis was performed using the LAS X software (Leica, Version 3.5.7.23225).

### 4.11. Gel Electrophoresis and Western Blotting

Following the re-circulation experiments, bound proteins were eluted from the washed adsorbent beads by incubation with Laemmli sample buffer (Bio-Rad, Vienna, Austria) at 95 °C for 5 min. The protein concentration was assessed using the DC Protein Assay (Bio-Rad), and protein extracts (20 μg protein per lane) were resolved by SDS-PAGE on 4–20% gels (Mini-PROTEAN TGX, Bio-Rad; Tris buffer, Bio-Rad) under reducing conditions and blotted onto nitrocellulose membranes. Membranes were incubated with anti-PF4 antibody (Santa Cruz Biotechnology) at a final concentration of 0.1 µg/mL and developed using the WesternBreeze Chemiluminescent Kit (Invitrogen, Waltham, MA, USA). Recombinant human carrier-free PF4 (R&D Systems), as well as the activated platelet concentrate used in the re-circulation experiments, were used as controls. Full gel is available in the Supplementary Figure S3.

### 4.12. Statistical Analysis

Statistical analysis was carried out using GraphPad Prism version 7.02 (La Jolla, CA, USA). Differences in the depletion of PF4, histones/nucleosomes and HMGB1 by the adsorbents, as well as concentration-dependent TRAP-6 activation of platelets in comparison to the controls, were analyzed by the non-parametric Kruskal–Wallis test followed by Dunn’s multiple comparisons test. Repeated-measure (RM) two-way ANOVA, followed by Sidak’s multiple comparisons tests, was performed to compare the effect of Heparin Sepharose and non-functionalized Sepharose at individual time points (30, 60 and 120 min). Data are presented as mean ± range or standard deviation. For all statistical tests, a value of *p* < 0.05 was considered statistically significant.

## Figures and Tables

**Table 1:** Adsorbent characteristics.

Adsorbent	Polymer	Mean Particle Size [µm]	Ligand Density	Supplier
Seraph-100	Polyethylene	370	Porcine heparin 2 mg/g beads	ExThera Medical, Martinez, CA, USA
Heparin Sepharose	Cross-linked agarose	90	Porcine heparin 2 mg/mL beads	Cytiva, Uppsala, Sweden
Sepharose	Cross-linked agarose	90	none	Cytiva, Uppsala, Sweden

**Table 2:** Antibodies and fluorochrome conjugates used for flow cytometry and confocal microscopy.

**Flow Cytometry**						
**Antigen**	**Origin**	**Clone**	**Marker for**	**Fluorochrome**	**Abbreviation**	**Supplier**
CD41	Mouse	P2	Platelets	Phycoerythrin Cyanin 7	PC7	Beckman Coulter
CD62P	Mouse	CLB Thromb6	Activated platelets	Fluorescein Isothiocyanate	FITC	Beckman Coulter
CD63	Mouse	H5C6	Activated platelets	Alexa Fluor 647	AF647	BioLegend
PF4	Mouse	# 170138	Activated platelets	Phycoerythrin	PE	R&D Systems
Anx5	-	-	Phosphatidyl-serine	Allophyco-cyanin	APC	BD Biosciences
**Confocal Microscopy**						
**Antigen**	**Origin**	**Clone**	**Marker for**	**Fluorochrome**	**Abbreviation**	**Supplier**
PF4	Mouse	D7	Activated platelets	-	-	Santa Cruz Biotechnology
Mouse IgG	Goat	Polyclonal	-	AlexaFluor 488	AF488	Jackson Immunoresearch

## Data Availability

All authors confirm that all relevant data are included in the manuscript. Additional statistical data are available from the corresponding author upon request. Data supporting the findings of this study are provided as supplementary information accompanying this paper.

## Supplementary Materials

The following supporting information can be downloaded at: https://www.mdpi.com/article/10.3390/ijms23031823/s1.
