# Supplementary material for: Heparin-Functionalized Adsorbents Eliminate Central Effectors of Immunothrombosis, including Platelet Factor 4, High-Mobility Group Box 1 Protein and Histones
Source: Int J Mol Sci. 2022 Feb 5;23(3):1823. doi: 10.3390/ijms23031823 (PMC8836755; doi:10.3390/ijms23031823)
Supplement: Supplementary file 1 [file ijms-23-01823-s001.zip › ijms-1542777-supplementary.pdf]

Supplementary Figures

Heparin-functionalized adsorbents eliminate central effectors of immunothrombosis, including platelet factor 4, high-mobility group box 1 protein, and histones

Marie Ebeyer-Masotta,[1] Tanja Eichhorn,[1] René Weiss,[1] Vladislav Semak,[1] Lucia Lauková,[1] Michael B. Fischer,[1,2] Viktoria Weber[1*]

1 Center for Biomedical Technology, Department for Biomedical Research, Danube University Krems, 3500 Krems, Austria

2 Clinic for Blood Group Serology and Transfusion Medicine, Medical University of Vienna, 1090 Vienna, Austria

*Correspondence: viktoria.weber@donau-uni.ac.at; phone: +43 2732 893 2601

Supplementary Figure S1

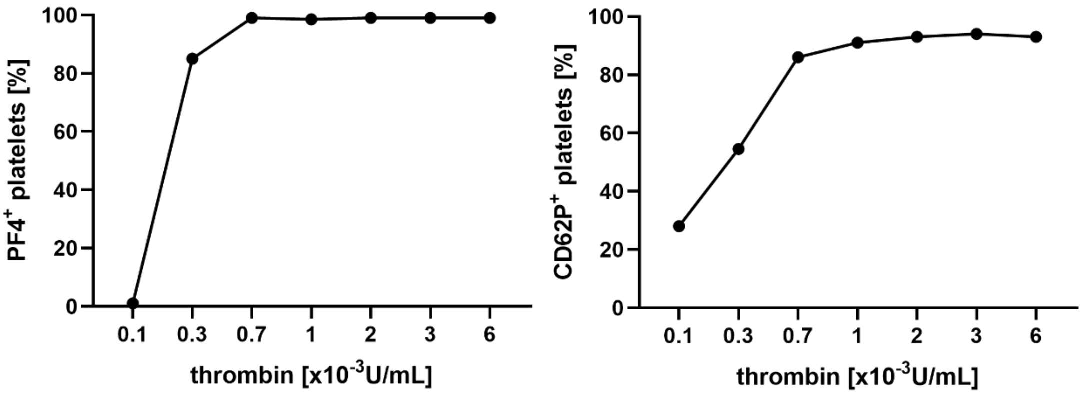

Supplementary Figure S1. Platelet activation with thrombin. Platelet-rich plasma was prepared from human whole blood anticoagulated with ethylene diamine tetraacetic acid. Whole blood was centrifuged at 500 g for 15 min at room temperature to obtain platelet-rich plasma (PRP). PRP was centrifuged at 800 g for 15 min at room temperature to pellet the platelets. Platelet pellets were resuspended in phosphate-buffered saline at a concentration of 10^7 platelets per ml and platelets were activated with increasing concentrations of thrombin (0.1, 0.3, 0.7, 1, 2, 3, 6 x 10^{-3} U/ml) for 10 min. Platelet activation was analyzed by flow cytometry as described in the main text, using CD41 as platelet marker and CD62P (P-selectin) as well as platelet factor 4 (PF4) as platelet activation markers.

Supplementary Figure S2

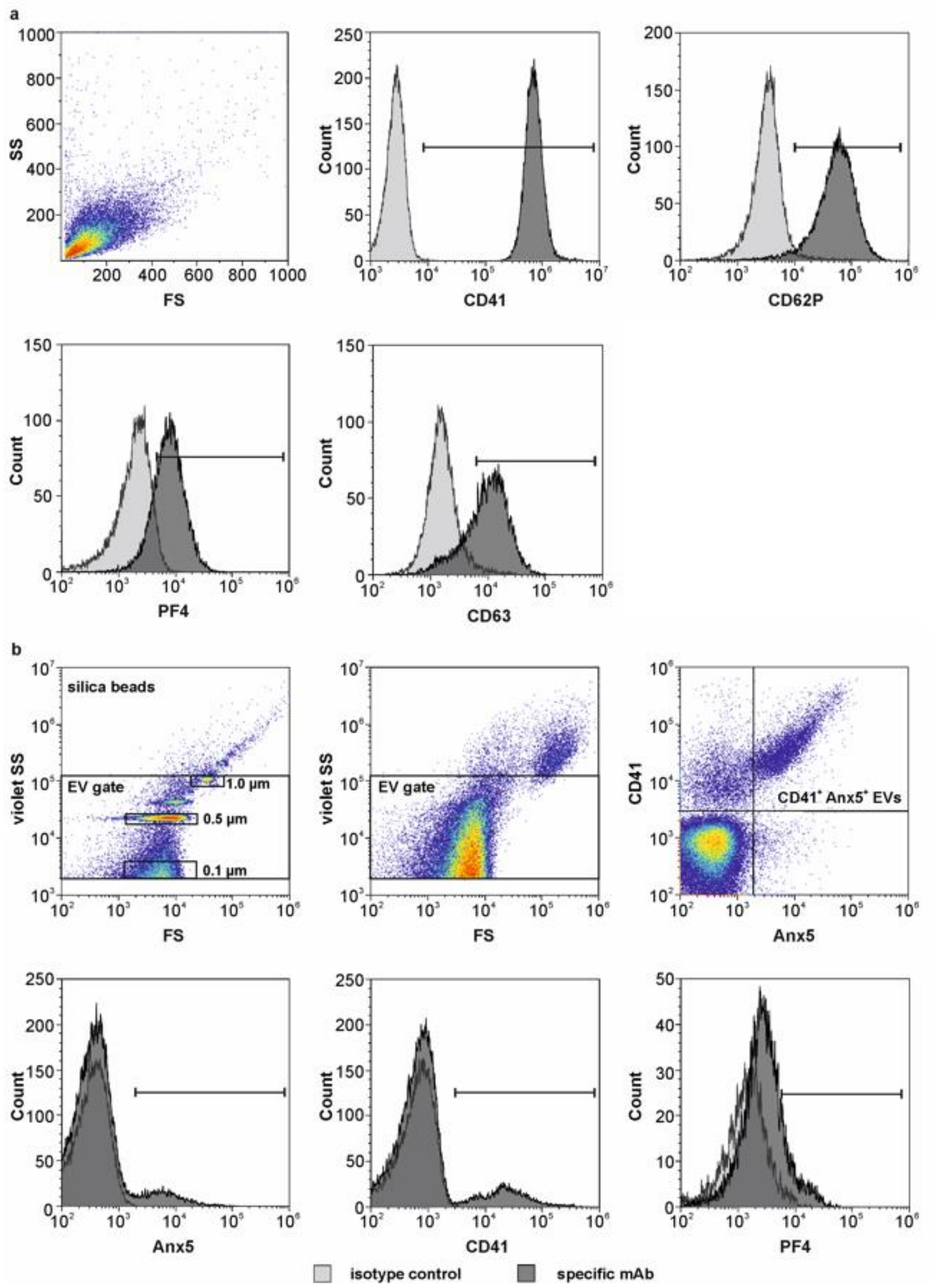

Supplementary Figure S2. Control experiments for the flow cytometric characterization of platelets and platelet-derived extracellular vesicles (pEVs). (**a**) For the analysis of platelets, the triggering signal was set to the 488 nm side scatter (SS). Platelet activation was monitored by using CD41 as platelet marker and CD62P, platelet factor 4 (PF4), and CD63 as platelet activation markers, as described in the main text. (**b**) For the characterization of pEVs, the triggering signal was set to the violet side scatter and the flow cytometer was calibrated with fluorescent green silica beads (1 µm, 0.5 µm, 0.3 µm, 0.1 µm; excitation/emission 485/510 nm). Staining of pEVs was performed with Annexin5 (Anx5) as marker for pEVs exposing phosphatidylserine in combination with CD41 as platelet marker and PF4 as platelet activation marker. In the case of Anx5, which binds to phosphatidylserine in a Ca^{2+}-dependent manner, staining was performed in the presence and absence of Ca^{2+}. The respective isotype controls and single stainings are shown. Bars indicate positive expression.

Supplementary Figure S3

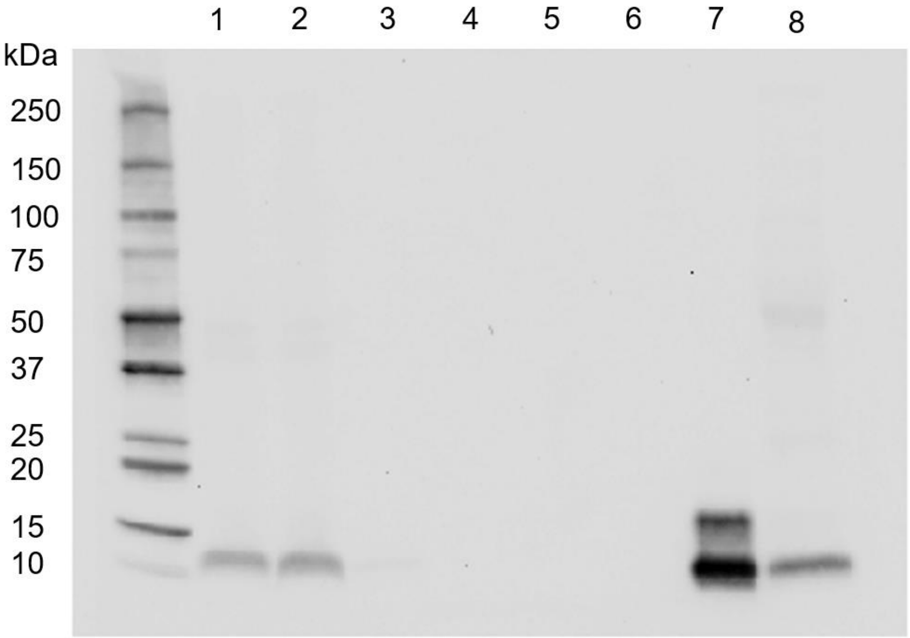

Supplementary Figure S3. Detection of PF4 by Western blotting. After 120 min of re-circulation of activated platelets over Heparin Sepharose or non-functionalized Sepharose, columns were opened, and adsorbent beads were collected. Proteins were eluted as described in detail in the main manuscript and analysed by Western blotting. Lanes 1-2, eluate from Heparin Sepharose; lanes 3-6, eluate from Sepharose; lane 7, human recombinant PF4; lane 8, activated platelet concentrate. 20 µg of protein were loaded per lane.
